# Predictors of Comorbid Anxiety Symptoms After a New Diagnosis of Epilepsy: A Prospective 12-Month Follow-Up Observation

**DOI:** 10.3389/fneur.2021.743251

**Published:** 2021-11-02

**Authors:** Rui Zhong, Weihong Lin, Qingling Chen, Xinyue Zhang, Guangjian Li

**Affiliations:** ^1^Department of Neurology, The First Hospital of Jilin University, Changchun, China; ^2^Department of Hepatology, Second People's Clinical College of Tianjin Medical University, Tianjin, China

**Keywords:** epilepsy, 12-month follow-up, depressive symptoms, anxiety symptom, number of ASMs

## Abstract

**Objectives:** We aimed to identify the factors contributing to comorbid anxiety symptoms over a 12-month follow-up period in Chinese adults with newly diagnosed epilepsy.

**Methods:** Adult patients with newly diagnosed epilepsy (PWNDE) were recruited from First Hospital, Jilin University. Anxiety symptoms were assessed using the Generalized Anxiety Disorder-7 questionnaire (GAD-7; Chinese version) at 12 months. Multivariate stepwise logistic regression analysis was employed to identify the predictors for anxiety symptoms at 12 months.

**Results:** A total of 157 PWNDE completed the study and were included in the final analysis. The percentage of participants with anxiety symptoms significantly decreased from 31.2% at baseline to 23.6% at 12 months (*p* = 0.027). Multivariate stepwise logistic regression analysis indicated that depressive symptoms at baseline [odds ratio (OR) 3.877 (95% confidence interval (CI) 1.683–8.933); *P* = 0.001] and the number of antiseizure medications (ASMs) during the follow-up period [OR 2.814 (95% CI 1.365–5.803); *P* = 0.005] were independent factors contributing to comorbid anxiety symptoms at 12 months.

**Conclusion:** Depressive symptoms at baseline and the number of ASMs during the follow-up period were significant predictors of comorbid anxiety symptoms 12 months after a diagnosis of epilepsy.

## Introduction

Epilepsy is a common severe brain disease that affects more than 70 million individuals worldwide (1, 2). Epilepsy rarely stands alone, and patients with epilepsy (PWE) always have one or several additional comorbidities (3–5). Epilepsy tends to be linked to psychiatric comorbidities, such as mood, anxiety, and psychotic disorders (6–9). Anxiety is a highly prevalent psychiatric comorbidity in PWE, and the incidence of anxiety is similar to that of depression (10–13). Among various populations, a total of 5–52.1% of PWE reported anxiety (14). Anxiety has been identified as a risk factor for poor quality of life, suicide risk, and poor seizure control in patients (15–18). It is of vital importance to identify the risk factors contributing to anxiety symptoms to improve preventive strategies. Physicians would benefit from information regarding which patients may be at higher risk for anxiety symptoms.

Although multiple studies aimed to identify the risk factors, including demographics and clinical characteristics, for anxiety in PWE, their findings were controversial (10, 11, 14, 19, 20). Female gender and depression were the most consistent risk factors associated with comorbid anxiety in PWE across studies. However, most of the previous studies employed a cross-sectional study design and were based on patients with chronic epilepsy. One major limitation is that this study design did not allow us to establish specific causal interpretations. Lee et al. recently reported that higher levels of anxiety symptoms at 1 year after the diagnosis of epilepsy could be predicted by higher neuroticism, stigma, and lower self-esteem (21). To date, there have been limited data investigating the risk factors for the development of anxiety symptoms in PWNDE. In this study, we aimed to identify the factors contributing to comorbid anxiety symptoms over a 12-month follow-up period in Chinese adults with newly diagnosed epilepsy.

## Methods

### Subjects

We conducted a prospective cohort study of PWNDE managed via an epilepsy management programme at the Epilepsy Clinic of First Hospital, Jilin University in Jilin Province. Adult PWNDE treated and followed up in our hospital between March 2017 and April 2020 were invited to participate in the current study. The diagnosis of epilepsy by a neurologist conformed to the International League Against Epilepsy (ILAE) criteria (22). Participants had never been treated with an ASM. The additional inclusion criteria were as follows: (1) 18 years of age or older; (2) physical, mental, and language abilities to complete the interview and questionnaires; and (3) willingness to participate. Exclusion criteria were as follows: (1) <18 years old; (2) a history of non-epileptic seizures; (3) a severe brain disease other than epilepsy (e.g., dementia and Parkinson's disease), a serious physical disease (e.g., significant hepatic, renal, or cardiopulmonary condition), or a psychiatric disorder (e.g., schizophrenia or lifelong anxiety); and (4) a history of intellectual disability or language disability. Written informed consent was obtained from all participants or their legal representatives. This study was approved by the Ethics Committee of First Hospital, Jilin University.

### Data Collection

Demographic and clinical variables were collected and recorded by a face-to-face structured interview at the time of diagnosis. We recorded demographic data, such as age, sex, marital status, educational level, occupational status, residence, and per capita monthly family income. Clinical variables [e.g., age at seizure onset, duration >6 months before diagnosis, seizure type, presence of generalized tonic-clonic seizure (GTCS) before diagnosis, and total of 5 or more seizures] were also obtained. Seizure type was classified as generalized, focal or unclassified onset. Formal follow-up outcome assessments were undertaken at 3, 6, and 12 months after enrolment. At each visit, patients and their relatives were questioned regarding seizure recurrence, medication compliance, and changes. Seizure-related variables during the 12-month follow-up, including the number of seizures and number of ASMs, were recorded. At the time of diagnosis (baseline), depressive and anxiety symptoms were assessed using the Neurological Disorders Depression Inventory for Epilepsy (NDDI-E; Chinese version) (23) and the 7-item Generalized Anxiety Disorder-7 questionnaire (GAD-7; Chinese version) (24), respectively. Anxiety symptoms were reassessed at the end of the 12-month follow-up.

### Questionnaires

#### Assessment of Anxiety and Depressive Symptoms

We adopted the Chinese version of the GAD-7 to assess anxiety symptoms in PWE (24). This instrument was validated for Chinese PWE with a suggested cut-off point of >6, with a sensitivity of 94% and a specificity of 91.4% (24). Cronbach's alpha was good for the Chinese version of the GAD-7 (alpha = 0.888). This questionnaire has seven self-rated questions, with each score ranging from zero to three (25). A continuous anxiety symptom severity score ranging from 0 to 21 was calculated. A higher GAD-7 score indicated more severe anxiety symptoms. The GAD-7 total score accurately distinguished those who had anxiety symptoms (GAD-7 total score >6) from those without anxiety symptoms (GAD-7 ≤6) (24).

The Chinese version of the NDDI-E (C-NDDI-E) scale is a rapid and user-friendly test used to evaluate depressive symptoms, which indicates the possibility of having comorbid depressive symptoms in PWE over the past 2 weeks (23). This questionnaire was validated for Chinese PWE with a suggested cut-off point of >12, with a sensitivity of 0.926 and a specificity of 0.804. Cronbach's alpha was good for the C-NDDI-E (alpha = 0.825) (23). The NDDI-E consists of a total of six self-rated questions, with each question offering four possible answers, which are scored from 1 to 4 points, generating a total score from 6 to 24 points, with higher scores indicating higher levels of depressive symptoms (26). A cut-off score of >12 indicates depressive symptoms in PWE (23). The NDDI-E and GAD-7 are not substitutes for clinical interviews and the Diagnostic and Statistical Manual (4th ed.) diagnosis, but they are reliable and validated self-report measures of anxiety and depressive symptoms in PWE, which have been widely used in mainland China (11, 14, 27).

### Statistical Analysis

The dependent variable was the presence or absence of comorbid anxiety symptoms at the end of the 12-month follow-up. Independent variables included characteristics at baseline and during the follow-up period. To confirm the factors contributing to the comorbid anxiety symptoms at 12 months, anxiety and depressive symptoms at baseline were also included as independent variables. Continuous data are presented as the means ± standard deviations (SDs) or medians [interquartile ranges (IQRs)] depending on the normal or non-normal distribution of the data assessed with the Kolmogorov-Smirnov test. Continuous variables were compared by Student's *t*-tests or Mann-Whitney *U*-tests. Categorical data are displayed as numbers with percentages and were compared by chi-squared tests or Fisher's exact tests. The Wilcoxon rank sum test and McNemar test were used to compare anxiety symptoms between baseline and 12 months after a diagnosis of epilepsy. Using multivariate analyses, the independent contribution of the study variables was assessed. Variables with a *P* < 0.05 in the univariate analyses were subsequently included in the multivariate stepwise logistic regression analysis. Receiver operating characteristic (ROC) curve analysis was utilized to test the overall prognostic accuracy of the significant predictors of anxiety symptoms at 12 months. The area under the curve (AUC) was calculated. All data were analyzed with SPSS 25.0 (SPSS Inc., Chicago, IL, USA). A probability value of *p* ≤ 0.05 was considered statistically significant.

## Results

### Demographic and Clinical Characteristics

A total of 157 PWNDE completed the study at the end of the 12-month follow-up period and were included in the final analysis. In this sample, 66 (42%) patients were women. The participants had a mean age of 35.34 ± 14.24 years. The demographic and clinical information of the participants is described in Table 1. Of the 157 PWNDE, 61.8% of them had a disease duration >6 months before diagnosis, and 48.4% of them had 5 or more seizures before treatment with ASMs. A total of 39 (24.8%) patients had depressive symptoms (C-NDDI-E >12), and 39 (31.2%) patients had anxiety symptoms (GAD-7 score >6) at the time of epilepsy diagnosis. Additionally, 62 (39.5%) patients experienced more than one epileptic seizure, and 22.9% of the patients were taking two or more ASMs during the follow-up period.

**Table 1:** Patient characteristics.

**Variable**	**Individuals who completed the study (*n* = 157)**
**Sociodemographic variables**	
Age (years), mean ± SD	35.34 ± 14.24
Female, *n* (%)	66 (42.0)
**Educational level**, ***n*** **(%)**	
University and above	45 (28.7)
Middle school	102 (65.0)
Primary school and below	10 (6.4)
Marital status-married, *n* (%)	101 (64.3)
Unemployed, *n* (%)	36 (22.9)
Residence-rural area, *n* (%)	59 (37.6)
**Per capita monthly family income (Yuan)**, ***n*** **(%)**	
<1,000	21 (13.4)
1,000–5,000	108 (68.8)
>5,000	28 (17.8)
**Seizure-related variables at baseline**	
Age at onset (years), median (IQR)	29 (18, 42)
Duration >6 months before diagnosis, *n* (%)	97 (61.8)
**Seizure type**, ***n*** **(%)**	
Focal	119 (75.8)
Generalized	24 (15.3)
Unclassified	14 (8.9)
Presence of GTCS before diagnosis, *n* (%)	98 (62.4)
Number of 5 or more seizures, *n* (%)	76 (48.4)
**Depression and anxiety symptoms at baseline**	
C-NDDI-E >12, *n* (%)	39 (24.8)
GAD-7 >6, *n* (%)	39 (31.2)
**Seizure-related variables at 12 months follow-up**	
**Number of seizures**, ***n*** **(%)**	
0	95 (60.5)
1–11	32 (20.4)
≥12	30 (19.1)
**Number of ASMs**, ***n*** **(%)**	
1	121 (77.1)
2	32 (20.4)
≥3	4 (2.5)

*GTCS, generalized tonic–clonic seizures; C-NDDI-E, Chinese version of Neurological Disorders Depression Inventory for Epilepsy; GAD-7, Generalized Anxiety Disorder-7 questionnaire; ASMs, antiseizure medications; SD, standard deviation; IQR, interquartile range; n, number*.

### Prevalence of Anxiety Symptoms at Baseline and at 12 Months

At the time of diagnosis, 31.2% of the 157 patients had comorbid anxiety symptoms. At the end of the 12-month follow-up period, the percentage of participants with anxiety symptoms significantly decreased from 31.2% at baseline to 23.6% at 12 months (*p* = 0.027; Table 2). Additionally, the median GAD-7 score significantly decreased from 4 at baseline to 3 at 12 months (*p* = 0.005).

**Table 2:** Comparison of anxiety symptoms between baseline and 12 months after a diagnosis of epilepsy (*n* = 157).

	**At baseline**	**At 12 months**	***P*-value**
GAD-7 score, median (IQR)	4 (1, 8)	3 (0, 6)	0.005
GAD-7 >6, *n* (%)	49 (31.2)	37 (23.6)	0.027

*GAD-7, Generalized Anxiety Disorder-7 questionnaire; IQR, interquartile range; n, number*.

### Factors Associated With Anxiety Symptoms at 12 Months

Univariate analyses indicated that comorbid anxiety symptoms at 12 months were significantly associated with depressive symptoms (*p* = 0.003) and anxiety symptoms (*p* = 0.027) at baseline (Table 3). Additionally, the number of seizures (*p* = 0.017) and number of ASMs (*p* = 0.041) during the follow-up period were risk factors contributing to comorbid anxiety symptoms at 12 months after diagnosis. Per capita monthly family income also tended to be associated with comorbid anxiety symptoms at 12 months, but the association did not reach statistical significance (*p* = 0.057). There was no significant difference between those with and without anxiety symptoms at 12 months in terms of demographic or seizure-related variables at baseline.

**Table 3:** Comparison of patient characteristics between patients with and without anxiety symptoms at 12 months.

**Variable**	**Anxiety symptoms at 12 months follow-up**		
	**Yes (*n* = 37)**	**No (*n* = 120)**	***P*-value**
**Sociodemographic variables at baseline**			
Age (years), mean ± SD	35.41 ± 15.04	35.32 ± 14.06	0.974
Female, *n* (%)	18 (48.6)	48 (40.0)	0.351
**Educational level**, ***n*** **(%)**			
University and above	9 (24.3)	36 (30.0)	0.442
Middle school	25 (67.6)	77 (64.2)	
Primary school and below	3 (8.1)	7 (5.8)	
Marital status-Married, *n* (%)	22 (59.5)	79 (65.8)	0.479
Unemployed, *n* (%)	27 (73.0)	94 (78.3)	0.498
Residence-rural area, *n* (%)	15 (40.5)	44 (36.7)	0.671
**Per capita monthly family income (Yuan)**, ***n*** **(%)**			
<1,000	9 (24.3)	12 (10.0)	0.057
1,000–5,000	23 (62.2)	85 (70.8)	
>5,000	5 (13.5)	23 (19.2)	
**Seizure-related variables at baseline**			
Age at onset (years), median (IQR)	28 (18, 42)	30 (19, 43)	0.532
Duration >6 months before diagnosis, *n* (%)	23 (62.2)	74 (61.7)	0.957
**Seizure type**, ***n*** **(%)**			
Focal	28 (75.7)	91 (75.8)	0.805
Generalized	6 (16.2)	18 (15.0)	
Unclassified	3 (8.1)	11 (9.2)	
Presence of GTCS before diagnosis, *n* (%)	27 (73.0)	71 (59.2)	0.13
Number of 5 or more seizures, *n* (%)	18 (48.6)	58 (48.3)	0.973
**Depression and anxiety symptoms at baseline**			
C-NDDI-E >12, *n* (%)	16 (43.2)	23 (19.2)	0.003
GAD-7 >6, *n* (%)	17 (45.9)	32 (26.7)	0.027
**Seizure-related variables at 12 months follow-up**			
**Number of seizures**, ***n*** **(%)**			
0	15 (40.5)	80 (66.7)	0.017
1–11	11 (29.7)	21 (17.5)	
≥12	11 (29.7)	19 (15.8)	
**Number of ASMs**, ***n*** **(%)**			
1	23 (62.2)	98 (81.7)	0.041
2	12 (32.4)	20 (16.7)	
≥3	2 (5.4)	2 (1.7)	

*GTCS, generalized tonic–clonic seizures; C-NDDI-E, Chinese version of Neurological Disorders Depression Inventory for Epilepsy; GAD-7, Generalized Anxiety Disorder-7 questionnaire; ASMs, antiseizure medications; SD, standard deviation; IQR, interquartile range; n, number*.

### Independent Factors Contributing to Anxiety Symptoms at 12 Months

Variables with a *P* < 0.05 in the univariate analyses were included in the multivariate stepwise logistic regression analysis. Multivariate stepwise logistic regression analysis indicated that depressive symptoms at baseline [odds ratio (OR) 3.877 (95% confidence interval (CI) 1.683–8.933); *P* = 0.001] and the number of ASMs during the follow-up period [OR 2.814 (95% CI 1.365–5.803); *P* = 0.005] were independent risk factors contributing to comorbid anxiety symptoms at 12 months (Table 4).

**Table 4:** Multiple logistic regression for predictors of anxiety symptoms at the end of the 12-month follow-up period.

**Variable**	**Anxiety symptoms at 12 months follow-up**		
	**OR**	**95% CI**	***P*-value**
**Depression and anxiety symptoms at baseline**			
C-NDDI-E >12, *n* (%)	3.877	1.683–8.933	0.001
**Seizure-related variables at 12 months follow-up**			
Number of ASMs	2.814	1.365–5.803	0.005

*C-NDDI-E, Chinese version of Neurological Disorders Depression Inventory for Epilepsy; ASMs, antiseizure medications; OR, odds ratio; CI, confidence interval*.

### The Predictive Value of Depressive Symptom Levels and the Number of ASMs for Anxiety Symptoms at 12 Months

**Figure 1:**
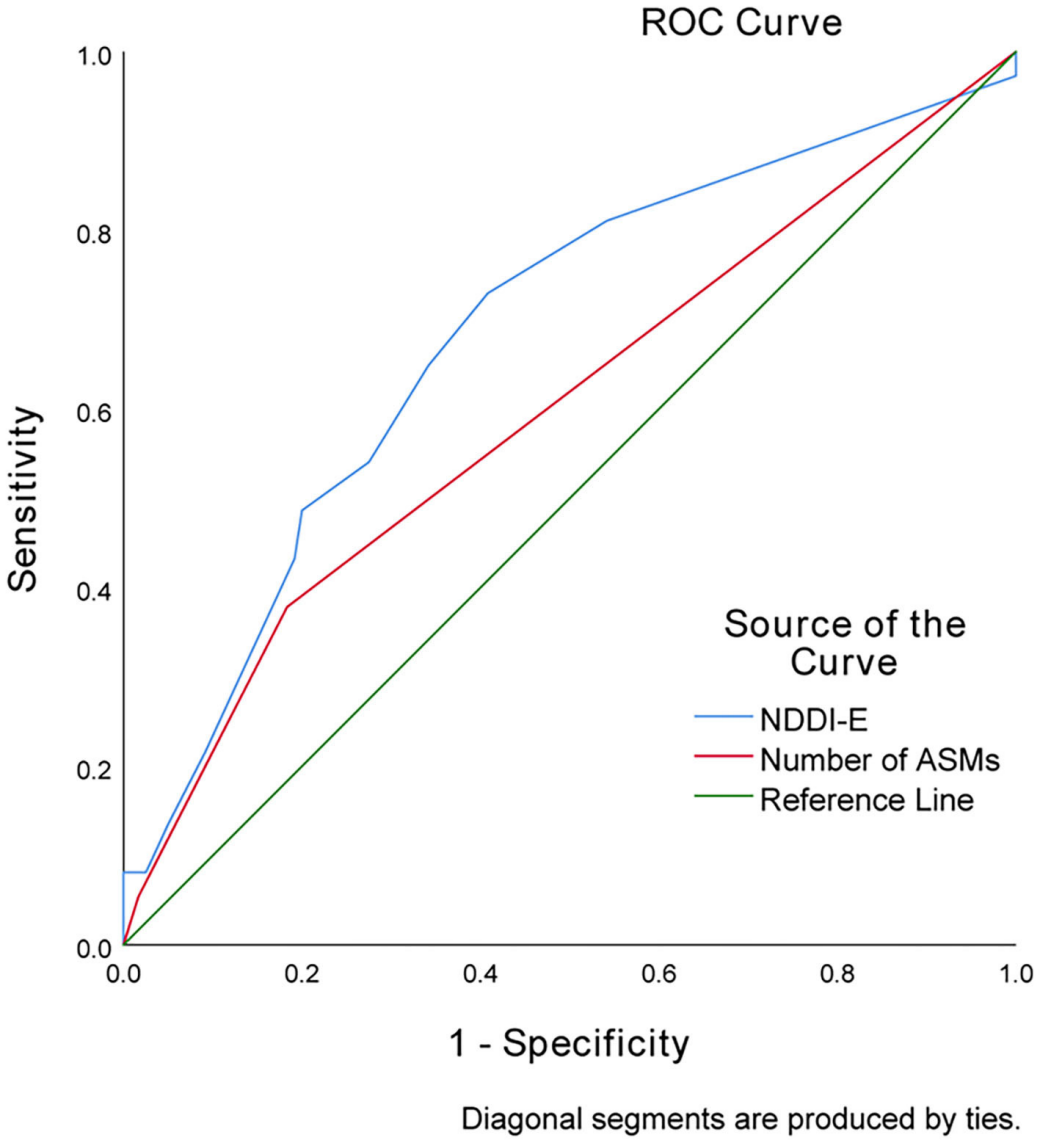
Receiver operator characteristic curve demonstrating sensitivity as a function of 1-specificity for predicting anxiety symptoms based on the NDDI-E scores and number of ASMs in PWNDE.

With an AUC of 0.684 (95% CI 0.583–0.785), depressive symptom scores (NDDI-E scores) showed a significantly greater discriminatory ability compared with that of the number of ASMs (AUC 0.599; 95% CI 0.490–0.709) to predict anxiety symptoms at 12 months (Figure 1).

## Discussion

Multiple cross-sectional studies of comorbid anxiety symptoms have been conducted on patients with chronic epilepsy (10, 14, 20, 28), but limited evidence has investigated the predictors for the development of anxiety symptoms in PWNDE (21). We aimed to identify the risk factors contributing to anxiety symptoms after a diagnosis of epilepsy. There were two main findings in this study. First, the incidence of comorbid anxiety symptoms decreased from 31.2% at baseline to 23.6% at the end of the 12-month follow-up period. Second, depressive symptoms at baseline and the number of ASMs during the follow-up period were independent risk factors for comorbid anxiety symptoms after 12 months of a diagnosis of epilepsy.

In this cohort, 31.2% of PWNDE had anxiety symptoms at baseline, which is lower than the 44.7% reported by Lee et al. in Korean adults with new-onset epilepsy using the Hospital Anxiety Depression Scale (HADS) (21). A prospective cohort study from Australia on PWNDE reported that anxiety was prevalent in 29% of the total participants at the time of diagnosis (29), which is similar to our reported incidence of comorbid anxiety symptoms. Notably, this may represent cross-cultural differences across studies. Another possible explanation is that the screening instruments for anxiety symptoms varied among studies. In this cohort, we also found that anxiety symptoms were prevalent in 31.2% of PWNDE at baseline, and they decreased over the follow-up period. These symptoms remained in 23.6% of patients at 12 months. A similar decrease in the incidence of anxiety over time was also reported by Lee et al. (21). In a recent prospective study from Australia with the aim of assessing mood trajectories after a first seizure, Velissaris et al. (30) found that anxiety trajectories decreased over time, and a patient's sense of poor control early after diagnosis was the main predictor of anxiety trajectories (31). Even decreasing anxiety trajectories over time were observed by prior investigations, and the influencing factor varied (21, 30). There may be cross-cultural differences in the major concerns of PWE in their daily life. For example, the most common concerns of adult PWE in West China were worries about seizures, maintaining a job, and the heritability of epilepsy (32). Anxiety/depression, age, and degree of discrimination were the main factors associated with the levels of concern in Korean patients with epilepsy (33).

In the present study, we revealed that the number of ASMs during the follow-up period was one of the most significant predictors of comorbid anxiety symptoms at 12 months. Our finding is in agreement with Oguz et al. (34) and Williams et al. (35), and they identified polytherapy as a significant risk factor for anxiety in children and adolescents with epilepsy (8). Anxiety has been strongly associated with the adverse side effects of ASMs (36, 37). The use of polytherapy may lead to more adverse effects from medications, which may contribute to anxiety in PWE (38). However, a 12-month follow-up study of 98 adults with new-onset epilepsy from South Korea reported that polytherapy was not a risk factor contributing to higher levels of anxiety symptoms (21). Additionally, the negative effects of some ASMs, such as phenobarbital, on mood have been identified (39). Data on the type of ASMs prescribed in PWE were not reported by those investigations, which may partly explain the controversial results.

This study also suggested that depressive symptoms at baseline emerged as another important predictor of anxiety symptoms at 12 months. In a prospective cohort study involving 439 individuals from Australia, Xu et al. provided evidence that a history of psychiatric disorder had a strong association with psychological distress in PWNDE (29). Additionally, it has been reported by recent cross-sectional studies that there may be a relationship between psychological distress and anxiety symptoms (29). Pre-pregnancy depression and/or anxiety was a risk factor associated with peripartum depression and/or anxiety in PWE (40). Prior evidence indicated that lifetime mood disorder was a predictor for seizure recurrence in adults with a single unprovoked seizure or newly diagnosed epilepsy (41). Similarly, higher levels of neuropsychiatric symptomatology were associated with a higher risk of seizure recurrence in patients newly treated with ASMs (42). Psychological distress appeared to persist due to seizure recurrence in the high anxiety group (30). Additionally, the mental health of PWE is significantly associated with the perceived stigma of the patients (43). A recent study from Italy showed that PWE were still affected by perceived stigma, which was strongly related to higher depressive symptoms (44). Thus, perceived stigma may be a significant predictor of anxiety symptoms, which requires more attention. Tombini et al. recently reported that the combination of depressive symptoms, perceived stigma, and the number of ASMs best explained the poor quality of life in PWE (45). Anxiety was a significant determinant of poor quality of life (16).

Several limitations exist for the current study. First, characteristics and follow-up data (e.g., number of seizures and educational levels) were gathered based on self-report. There is the possibility of the existence of self-report bias. Second, some variables that may contribute to the development of anxiety symptoms after a new epilepsy diagnosis (e.g., ASM type or family psychiatric history) were not available and were not analyzed in our cohort. Additionally, we do not have reliable information on counseling or psychological treatments. This variable was not included as a possible confounder, and its potential effects on our results were not assessed. Third, all participants were limited to adults with newly diagnosed epilepsy and recruited from a single centre in northeast China, which may have introduced selection bias. Thus, our findings may not be relevant to all patient groups.

## Conclusion

In conclusion, we found that the prevalence of comorbid anxiety symptoms decreased from 31.2% at baseline to 23.6% at 12 months in PWNDE. Additionally, depressive symptoms at baseline and the number of ASMs during the follow-up period were independent risk factors for comorbid anxiety symptoms after 12 months of diagnosis.

## Data Availability Statement

The raw data supporting the conclusions of this article will be made available by the authors, without undue reservation.

## Ethics Statement

The studies involving human participants were reviewed and approved by Ethics Committee of First Hospital, Jilin University. The patients/participants provided their written informed consent to participate in this study.

## Author Contributions

GL and WL conceived and designed the study. RZ, QC, and XZ were involved in data acquisition. RZ analyzed the data and wrote the manuscript. All authors contributed to the article and approved the submitted version.

## Funding

This work was supported by a grant from the Programme of Jilin University First Hospital Clinical Cultivation Fund (LCPYJJ2017006).

## Conflict of Interest

The authors declare that the research was conducted in the absence of any commercial or financial relationships that could be construed as a potential conflict of interest.

## Publisher's Note

All claims expressed in this article are solely those of the authors and do not necessarily represent those of their affiliated organizations, or those of the publisher, the editors and the reviewers. Any product that may be evaluated in this article, or claim that may be made by its manufacturer, is not guaranteed or endorsed by the publisher.
